# Specific molecular allergic sensitisation patterns in pediatric polysensitised patients

**DOI:** 10.1186/1939-4551-8-S1-A152

**Published:** 2015-04-08

**Authors:** Teresa Garriga Baraut, Cristina Blasco, Blanca Vilá, Karla Narváez, Antonio Moreno-Galdó, Victòria Cardona, Mercè Guillén, Elianett Pérez, Moisés Labrador-Horrillo

**Affiliations:** 1 Vall D'hebron Hospital, Spain

## Background

Advances in the characterization of allergens have allowed the development of new diagnostic tools based on purified and recombinant allergens. The complex information provided by these multiplexed systems needs a careful interpretation in the light of the local characteristics of patients. Therefore, there is a need to define the sensitisation profiles of each population. The aim of the study was to define specific molecular allergic sensitisation patterns in pediatric polysensitised patients from the Mediterranean area (Barcelona).

## Methods

Pediatric patients experiencing food symptoms (oral allergy syndrome, urticaria, angioedema, gastrointestinal symptoms or anaphylaxis) who were sensitised to two or more unrelated food groups (excluding milk and egg) by skin prick test (SPT) were included. These patients may or may not also have respiratory symptoms (rhinoconjunctivitis and/or asthma). SPT were performed with standardized food and inhalant allergen extracts. The following parameters were measured: total serum IgE, specific IgE (ImmunoCAP®, Thermofisher Scientific) to those allergens shown positive on the SPT, specific IgE to a panel of recombinant allergens by the commercial microarray ImmunoCAP-ISAC® (Thermofisher Scientific), version 112, that contains 112 individual components. Data analyses were performed using the SPSS Package (release 22.0).

## Results

120 patients were included (66 males) with an average age of 11 years (range 4-18). 73% of patients had a family history of allergy. Lipid transfer protein (LTP) was the most prevalent protein sensitisation in our population (74%), followed by storage proteins (57%), tropomyosin (40%) and parvalbumin (29%). Regarding LTP sensitisation, the most frequent molecule determined was Jug r 3 (69%), followed by Pru p 3 (67%) and Pla a 3 (63%), while Jug r 2 (42%), Jug r 1 (38%), Gly m 6 (31%) and Cor a 9 (29%) were the most frequent storage proteins sensitisations shown in our Mediterranean population. There was no statistical association between sensitization to Pru p 3 and anaphylaxis due to vegetable foods (p=0.37). However, as the patient’s age increases, the rate of anaphylaxis caused by vegetable foods rises (p<0.05).

## Conclusions

Poly-sensitised food-allergic pediatric patients from Barcelona are mostly sensitised to LTP, followed by storage proteins. However, in our population LTP and storage proteins are not associated with increases rates of anaphylaxis. Multiplexed molecular diagnosis delivers added information which may be useful in the management of these patients.

